# Preparation for action: Psychophysiological activity preceding a motor skill as a function of expertise, performance outcome, and psychological pressure

**DOI:** 10.1111/psyp.12182

**Published:** 2014-02-24

**Authors:** Andrew Cooke, Maria Kavussanu, Germano Gallicchio, Adrian Willoughby, David McIntyre, Christopher Ring

**Affiliations:** a School of Sport, Health & Exercise Sciences, Bangor UniversityBangor, UK; b School of Sport, Exercise & Rehabilitation Sciences, University of BirminghamBirmingham, UK

**Keywords:** EEG, Heart rate deceleration, Movement kinematics, Expertise, Performance under pressure

## Abstract

Knowledge of the psychophysiological responses that characterize optimal motor performance is required to inform biofeedback interventions. This experiment compared cortical, cardiac, muscular, and kinematic activity in 10 experts and 10 novices as they performed golf putts in low- and high-pressure conditions. Results revealed that in the final seconds preceding movement, experts displayed a greater reduction in heart rate and EEG theta, high-alpha, and beta power, when compared to novices. EEG high-alpha power also predicted success, with participants producing less high-alpha power in the seconds preceding putts that were holed compared to those that were missed. Increased pressure had little impact on psychophysiological activity. It was concluded that greater reductions in EEG high-alpha power during preparation for action reflect more resources being devoted to response programming, and could underlie successful accuracy-based performance.

Identifying the movement-related psychophysiological response patterns that characterize successful motor performance could yield important benefits for society. For instance, such knowledge could inform customized biofeedback training to expedite skill acquisition, and increase the likelihood of successful performance outcomes in any domain where steep learning curves and accurate motor performance are critical (e.g., medicine, armed forces, sport). To provide insight into psychophysiological responses associated with successful motor performance, researchers have typically analyzed measures of cardiac or electroencephalographic (EEG) activity during the final seconds preceding movements, with these measures being interpreted to reflect preparatory information processing and motor response programming (e.g., Lacey & Lacey, 1974; Pfurtscheller & Aranibar, 1979). Previous research has also assumed that for a movement-related psychophysiological response to characterize successful motor performance, it should distinguish experts from novices and successful from unsuccessful performance outcomes (e.g., Cooke, 2013; Hatfield, Haufler, Hung, & Spalding, 2004). The goal of this experiment was to extend previous research via pathways described in the following sections, and thereby provide new insight into the psychophysiology of optimal motor performance.

## Preparation for Action: Overview of Previous Research

One of the most consistent findings of previous preparation for action research comes from studies of golf putting. Specifically, research has shown that golf putts are preceded by a deceleration in heart rate, which is greater in experts than novices. For instance, Boutcher and Zinsser (1990) found that, from a premovement baseline, expert and novice golfers reduced their heart rate by 20 beats per minute (bpm), and 15 bpm, respectively, during the four interbeat intervals preceding 12-foot putts. Similarly, Neumann and Thomas (2009) found that elite, experienced, and novice golfers reduced their heart rate by 12, 10, and 2 bpm, respectively, during the 6 s preceding 8-foot putts.

These findings have been interpreted through Lacey and Lacey's sensory intake-rejection hypothesis (e.g., Lacey & Lacey, 1970, 1974, 1980). It contends that decelerations in heart rate facilitate external processing by reducing blood pressure and thereby unloading the baroreceptors to increase the flow of environmental information to the brain (Brunia, 1993). Accordingly, the greater magnitude of heart rate deceleration in experts in the moments preceding golf putts is thought to indicate that experts engage in more preparatory external information processing than novices (Neumann & Thomas, 2009).

However, in light of the aforementioned findings, it is surprising to note that research has yet to examine any differences in the magnitude of heart rate deceleration preceding holed versus missed putts. Such a difference would be expected to emerge (i.e., greater deceleration preceding holed putts) if preparatory heart rate deceleration is related to performance accuracy. This hypothesis has been examined by a handful of studies outside of golf, but the evidence associating heart rate deceleration with accuracy is mixed. For instance, Tremayne and Barry (2001) found a marginally significant linear trend, which indicated a steeper decline in the heart rate of expert pistol shooters in the 5 s preceding shots that hit the bulls-eye, when compared to shots that missed. However, Konttinen, Lyytinen, and Viitasalo (1998) found that preparatory heart rate decelerations had no effect on the shot outcomes of elite and experienced rifle shooters. Accordingly, the relationship between preparatory heart rate and performance warrants further scrutiny.

Research investigating patterns of EEG activity preceding putts is scarcer than the studies of heart rate, but two recent EEG studies are noteworthy. First, Baumeister, Reinecke, Liesen, and Weiss (2008) compared the EEG activity of expert and novice golfers. The results of this between-subjects investigation indicated that, while putting for 4 min, experts produced more frontal theta (4.75–6.75 Hz) and parietal high-alpha (9.75–12.5 Hz) power than novices. Based on the well-established view that EEG alpha power is inversely related to cortical activity (e.g., Pfurtscheller, 1992), Baumeister et al. (2008) interpreted their findings as evidence for expert-related neural efficiency: The greater accuracy of experts compared to novices was associated with an economy of effort, whereby experts were able to expend fewer neural resources than novices to achieve success.

Second, Babiloni et al. (2008) conducted a within-subjects analysis that compared patterns of EEG activity preceding holed putts and missed putts in a sample of expert golfers. First, they found a widespread reduction in EEG alpha power during the 4 s preceding putts. This is in line with the well-established finding that voluntary self-paced movements are preceded by a reduction (i.e., desynchronization) in EEG alpha power (around 8–12 Hz), which occurs in both hemispheres of the brain during bimanual tasks (e.g., Leocani, Toro, Manganotti, Zhuang, & Hallett, 1997; Pfurtscheller & Aranibar, 1979; Pfurtscheller & Lopes da Silva, 1999). Crucially, Babiloni et al. (2008) also found that, compared to missed putts, holed putts were preceded by a greater reduction in high-alpha power (10–12 Hz) at sites roughly overlying the premotor and motor cortex (e.g., Fz, Cz, C4). This finding could reflect greater concentration and more neural resources being devoted to movement programming ahead of putts that are holed.

While the findings of Babiloni et al. (2008) and Baumeister et al. (2008) appear to contrast, it should be noted that the measures of cortical activity obtained by Baumeister et al. (2008) were averaged over a 4-min recording block rather than being event-locked to individual putts. Accordingly, this study assessed only gross differences in activity, and, as such, potential dynamic expert and novice differences in movement-related EEG activity (i.e., activity occurring only in the seconds immediately preceding movement) could not be determined. To clarify the cortical profile of successful golf putting, research should therefore evaluate movement-related cortical activation as a function of both expertise (i.e., a between-subjects factor) and outcome (i.e., a within-subjects factor).

## Effects of Pressure on Movement-Related Psychophysiological Activity

Few studies have examined the effects of increased psychological pressure on movement-related psychophysiological activation. This is surprising because elevated levels of pressure are commonplace in many movement domains such as the armed forces and sport (Hatfield et al., 2004), with increased pressure often eliciting a strong influence on our behavior (e.g., Baumeister & Showers, 1986; Beilock & Gray, 2007). A handful of studies have indicated that increased pressure can augment tonic heart rate and muscle activity (e.g., Cooke, Kavussanu, McIntyre, & Ring, 2010), elongate the duration of muscle contractions (e.g., Weinberg & Hunt, 1976), and elicit more variable (less efficient) movement patterns (e.g., Pijpers, Oudejans, & Bakker, 2005). However, the effects of pressure on the patterns of movement-related heart rate and EEG activity described above are yet to be examined.

## The Present Study

Building on the literature reviewed above, the present study adopted a multifactorial design that allowed the comparison of both expertise (i.e., novice vs. expert) and performance outcome (i.e., holed putts vs. missed putts) on preparatory psychophysiological responses. We also conducted the first investigation of the effects of increased pressure on movement-related heart rate and EEG activity. Finally, to corroborate and extend previous pressure-based research as described above (i.e., Cooke et al., 2010; Pijpers et al., 2005; Weinberg & Hunt, 1976), we recorded movement kinematics and muscle activity. In doing so, our study was designed to paint the richest picture to date of how multiple systems (i.e., cardiac, cortical, muscular, and kinematic) operate to control motor performance under both pressure-free and pressure-laden conditions.

We hypothesized a series of interaction effects such that golf putts would be preceded by a deceleration in heart rate and a reduction in EEG high-alpha power that would be greater in experts than novices, and greater before holed compared to missed putts. Moreover, we expected increased pressure to elevate heart rate and muscle activity, perturb movement kinematics, and thereby impair performance (e.g., Cooke et al., 2010). Finally, if increased pressure was to have a detrimental effect on performance, we expected that this would be accompanied by reductions in the magnitude of preparatory heart rate deceleration and modified patterns of EEG activity to reflect more worrisome thoughts in the high-pressure condition (see Carter, Johnson, & Borkovec, 1986).

## Method

### Participants

Ten expert (*M* age = 20.90, *SD* = 0.74 years) and ten novice (*M* age = 19.00, *SD* = 0.66 years) right-handed male golfers volunteered to participate in the experiment. The experts had a mean of 11.25 (*SD* = 3.78) years of golf experience and were required to have a golf handicap < 5 (*M* handicap = 1.50, *SD* = 2.32). The novices had a mean of 1.85 (*SD* = 2.49) years of golf experience and had no formal golf handicap. All participants provided informed consent. The protocol was approved by the local research ethics committee.

### Task

In line with previous preparation for action research (e.g., Babiloni et al., 2008; Baumeister et al., 2008; Boutcher & Zinsser, 1990; Neumann & Thomas, 2009, 2011), we adopted a golf putting task. This task benefits from being fairly stationary in nature, providing readily available information concerning performance outcome, and from being a task that requires the accurate programming of both movement force and movement direction. Importantly, this latter benefit ensures that golf putting could resonate with tasks that have similar requirements in other less-accessible domains.

Participants used a standard length (90 cm) blade style golf putter (Scotty Cameron Circa 62, Titleist, Fairhaven, MA) to putt regular-sized (diameter 4.7 cm) golf balls towards a hole from a distance of 2.4 m. The hole was located 1.5 m from the end and 0.7 m from the side of a strip of artificial putting mat (Patiograss), and had a diameter of 10.8 cm (i.e., standard size) for novices and 5.4 cm (i.e., through placing a bespoke insert into the standard hole) for experts. This distance and two hole sizes were chosen following pilot testing to ensure a similar success rate for experts and novices, with at least 30% of all putts being missed. Ensuring a similar number of holed and missed putts was necessary in order for putt outcome to be included as a within-subjects factor in our analyses (e.g., Babiloni et al., 2008). Percentage of putts holed was assessed in order to ascertain whether this manipulation of hole diameter was a success.

### Design

We adopted a mixed multifactorial design, with group (novice, expert) as a between-subjects factor, and performance outcome (holed putts, missed putts), pressure (low, high), and epoch as within-subjects factors. Epoch refers to the time windows preceding movement during which each psychophysiological variable was assessed. For example, in line with two recent studies, heart rate and muscle activity were assessed in 13 epochs (i.e., −6 s, −5 s, −4 s, −3 s, −2 s, −1 s, 0 s, +1 s, +2 s, +3 s, +4 s, +5 s, +6 s) around movement initiation (e.g., Moore, Vine, Cooke, Ring, & Wilson, 2012; Neumann & Thomas, 2011). It was necessary to include this factor in our design in order to test the hypothesized interaction effects (e.g., that experts would produce a greater heart rate deceleration than novices). Further details of the epoch factor are provided in the data reduction and statistical analyses sections below.

### Measures

#### Cardiac activity

Cardiac activity was derived from an electrocardiogram (ECG) obtained using three silver/silver chloride spot electrodes (Cleartrace, ConMed, Utica, NY) in a modified chest configuration. The ECG signal was amplified (Bagnoli-4, Delsys, Boston, MA), filtered (1–100 Hz), and digitized at 2500 Hz with 16-bit resolution (Power 1401, Cambridge Electronic Design, Cambridge, UK) using Spike2 software (Cambridge Electronic Design).

#### Cortical activity

EEG activity was recorded from an array of 16 silver/silver chloride pin electrodes on the scalp (Fp1, Fp2, F4, Fz, F3, T7, C3, Cz, C4, T8, P4, Pz, P3, O1, Oz, O2) positioned in accordance with the 10–20 system (Jasper, 1958). Common mode sense (CMS) and driven right leg (DRL) electrodes were used to enhance the common mode rejection ratio of the EEG signals. Electrodes were also placed at the left and right mastoids, to permit offline referencing. All signals were amplified and digitized at 512 Hz with 24-bit resolution (ActiveTwo, BioSemi, Amsterdam, The Netherlands) using ActiView software (BioSemi).

#### Muscle activity

Muscle activity was derived from an electromyogram (EMG) measured using a differential surface electrode (DE 2.1, Delsys) affixed to the extensor carpi radialis of the left arm, and a ground electrode (Cleartrace) on the left collarbone. The left extensor carpi radialis was chosen based on previous research implicating this muscle in the putting stroke of right-handed golfers (e.g., Cooke et al., 2010; Cooke, Kavussanu, McIntyre, Boardley, & Ring, 2011). The EMG signal was amplified (Bagnoli-4, Delsys), filtered (20–450 Hz), and digitized at 2500 Hz with 16-bit resolution (Power 1401) using Spike2 software.

#### Movement kinematics

Movement kinematics were recorded using a triaxial accelerometer (LIS3L06AL, ST Microelectronics, Geneva, Switzerland). Acceleration on the X, Y, and Z axes corresponded to lateral, vertical, and back-and-forth movement of the clubhead, and assessed clubhead orientation, clubhead height, and impact velocity, respectively. The signals were conditioned by a bespoke buffer amplifier with a frequency response of DC to 15 Hz. Both accelerometer and amplifier were mounted in a 39 mm × 20 mm × 15 mm plastic housing secured to the rear of the putter head.

### Pressure Manipulation

Participants completed two blocks of putts designed to represent comparatively low- and high-pressure conditions. Pressure was manipulated using social evaluation, competition, and rewards (Baumeister & Showers, 1986). The pressure conditions were preceded by a cover story presented to participants in the pre-experiment briefing. Specifically, participants were informed that one aim of our study was to compare Titleist ProV1 and Titleist ProV1x golf balls. Accordingly, they were told that they would complete two blocks of putts, the first using a ProV1 golf ball, and the second using a ProV1x golf ball. These two blocks, which represented the low- and high-pressure conditions described next, were completed in an order that was counterbalanced across participants.

#### Low pressure

The low-pressure condition was a noncompetitive condition that capitalized on the cover story presented in the experiment briefing. Participants were informed that performance would be assessed by the percentage of putts holed, and the average distance of putts from the hole, with holed putts counting as 0 cm in this calculation. Crucially, participants were also told that, although the accuracy of each putt would be recorded, their individual performance would not be analyzed in this block. Instead, it was explained that the performance of all participants would be pooled to generate one accuracy score for whichever golf ball was being used (i.e., the ProV1 for participants who completed the low-pressure condition first, and the ProV1x for participants who completed the low-pressure condition second). In reality, we did not compare the two golf balls; this cover story was simply used to minimize any pressure that may have been elicited by evaluation from the experimenter in the low-pressure condition.

#### High pressure

We sought to maximize evaluation and competition in the high-pressure condition. Specifically, participants were told that, in addition to using the opposite ball (i.e., ProV1 or ProV1x) to the one they used/were going to use in the low-pressure condition, they would also be individually evaluated in this block of putts. To this end, they were told that all participants would be ranked on a leaderboard based on the percentage of putts that they holed in this condition. Moreover, if there was a tie on the percentage of putts holed, rankings would be determined by the average distance that putts finished from the hole. Finally, they were informed that the leaderboard would be e-mailed to all participants at the end of the study, and that cash prizes of £100, £50, and £30 would be awarded to the top three performers (e.g., Cooke et al., 2011).

### Manipulation Check

To establish the effectiveness of our manipulation in creating two distinct levels of pressure, participants completed the 5-item pressure/tension subscale of the Intrinsic Motivation Inventory (Ryan, 1982) immediately after each block of putts. Items, including “I felt pressured,” were rated on a 7-point Likert scale, with labels of 1 (*not at all true*), 4 (*somewhat true*), and 7 (*very true*). The item responses were averaged to provide one score for the subscale. Cooke et al. (2011) reported reliability coefficients ranging from .66 to .90 for this subscale. In this experiment, alpha coefficients were .73 in the low-pressure condition and .72 in the high-pressure condition, thereby demonstrating acceptable internal consistency.

### Procedure

Participants attended a single 2-h testing session. After being briefed and providing consent to participate, they were equipped with instruments to allow the recording of physiological measures, and provided with instructions about the golf putting task. Specifically, participants were asked to try to get all putts “ideally in the hole, but if unsuccessful, to make them finish as close to the hole as possible.” Next, they performed a block of 20 familiarization putts using a Dunlop DDH golf ball (i.e., a neutral ball not used in the pressure conditions) to become accustomed to the putting surface and to putting while instrumented for physiological recordings. After the familiarization block, participants performed two blocks of 60 putts, which represented the low- and high-pressure conditions. Each block was preceded by its respective pressure manipulation as described above. After each putt, a photograph was taken to record the terminal location of the ball (see Neumann & Thomas, 2008), and then the ball was replaced at the start position by the experimenter. This ensured that participants did not need to move between trials, thereby keeping movement artifacts to a minimum while also regulating the time between putts, which approximately ranged from 17–25 s.

The manipulation check was administered immediately after the final putt in each of the pressure conditions, while physiological measures were recorded continuously during each block. On completion of the session, participants were thanked, debriefed, and questioned regarding any ball preference (i.e., Titleist ProV1 vs. Titleist ProV1x). Their answers indicated that the low-pressure manipulation was credible; none guessed the true goal of this part of the experiment. Finally, on completion of the study, the leaderboards were e-mailed to participants, and competition winners were contacted and paid their prize money.

### Data Reduction

Individual trials within the continuous physiological recordings were identified using an optical sensor (S51-PA 2-C10PK, Datasensor, Monte San Pietro, Italy), which detected the initiation of putts, and a microphone (NT1, Rode, Silverwater, Australia) connected to a mixing desk (Club 2000, Studiomaster, Leighton Buzzard, UK), which detected the putter-to-ball contacts. These signals were recorded using both Actiview (BioSemi) and Spike2 software.

#### Cardiac and muscle activity

In line with two recent studies (i.e., Moore et al., 2012; Neumann & Thomas, 2011), we used the ECG and EMG signals to compute heart rate and muscle activity in successive 1-s epochs from 6 s before until 6 s after the initiation of each putt. Heart rate was derived from the intervals between the R-waves of the ECG. Muscle activity was calculated by rectifying the EMG signal and averaging over 500-ms windows, such that the mean activity between 6.25 and 5.75 s prior to movement was used to calculate muscle activity 6 s before movement, and so on (e.g., Moore et al., 2012).

#### Cortical activity

EEG signal processing was conducted through EEGLAB software (Delorme & Makeig, 2004) using the following procedure: First, data files were resampled (256 Hz), filtered (1–50 Hz), and referenced to the average mastoid. Next, we set about identifying a neutral EEG baseline (cf. Babiloni et al., 2008). Specifically, we performed a fast Fourier transform (1 Hz bins) spanning 7 s before until 1 s after the initiation of each putt. We then performed exploratory analyses to identify a period within this 8-s window where cortical activity was similar across both between- (i.e., group) and within-subjects factors (i.e., outcome and pressure). We identified a period from 4 s to 3 s before movement as the optimal baseline, before proceeding with the remaining data processing steps as follows: First, we created new epochs spanning 5 s before until 1 s after each putt (e.g., Babiloni et al., 2008), and performed baseline removal. Next, we screened the data to reject any artifacts. Gross artifacts were removed by rejecting fluctuations in the signal of greater than 100 μV. Remaining artifacts including eye blinks, eye movements, and the pulse were then identified and removed using independent component analyses (e.g., Delorme & Makeig, 2004) and the ADJUST algorithm (Mognon, Jovicich, Bruzzone, & Buiatti, 2011). Third, we performed a fast Fourier transform (1 Hz bins) on the artifact-free epochs, and averaged the data in successive 1-s epochs from 4 s before (i.e., preparatory period) until 1 s after (i.e., movement period) the initiation of putts. Finally, we computed power in theta (4–7 Hz), low-alpha (8–10 Hz), high-alpha (10–12 Hz), and beta (13–32 Hz) frequency bands.

For brevity of reporting, only the results of the Fz, F3, F4, Cz, C3, and C4 electrodes are presented. We selected these electrodes because they roughly overlie the primary motor cortex, the premotor cortex, and the supplementary motor areas that are related to movement control (e.g., Ashe, Lungu, Basford, & Lu, 2006) and which have been implicated in previous EEG-based golf putting research (Babiloni et al., 2008). Moreover, topographical analyses revealed that these electrode sites were largely representative of the others while capturing the strongest effects.

#### Movement kinematics

To compute kinematic variables, we scored acceleration for each putt from the onset of the downswing phase of the putting stroke until the point of ball contact (e.g., Cooke et al., 2010, 2011; Moore et al., 2012). Specifically, we calculated average acceleration for the X, Y, and Z axes, and impact velocity for the Z axis as the primary axis involved in the putting stroke.

### Statistical Analyses

Our analyses included group (expert, novice) as a between-subjects factor, and a combination of pressure (low, high), outcome (hit, miss), and epoch (physiological variables in successive 1-s epochs around putts) as within-subjects factors. Accordingly, our manipulation check variables, namely, percentage of putts holed and self-reported pressure, were subjected to 2 Group × 2 Pressure analyses of variance (ANOVAs). Our measures of cardiac and muscle activity were subjected to 2 Group × 2 Pressure × 2 Outcome × 13 Epoch (i.e., −6 s, −5 s, −4 s, −3 s, −2 s, −1 s, 0 s, +1 s, +2 s, +3 s, +4 s, +5 s, +6 s) ANOVAs. Our cortical measures were subjected to 2 Group × 2 Pressure × 2 Outcome × 5 Epoch (i.e., −4 to −3 s, −3 to −2 s, −2 to −1 s, −1 s to 0 s, 0 s to +1 s) ANOVAs. Finally, our kinematic measures were subjected to 2 Group × 2 Pressure × 2 Outcome ANOVAs. Significant effects were probed by polynomial trend analyses and planned post hoc comparisons. The results of univariate tests are reported, with the Huynh-Feldt correction procedure applied for analyses that violated the sphericity of variance assumption. Partial eta-squared is reported as a measure of effect size, with values of .02, .12, and .26 indicating relatively small, medium, and large effect sizes, respectively (Cohen, 1992).

## Results

### Manipulation Checks

#### Percentage of putts holed

A 2 Group (expert, novice) × 2 Pressure (low, high) ANOVA conducted on the percentage of putts holed revealed no significant effects: group, *F*(1,18) = 1.79, *p* = .20, 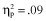; pressure, *F*(1,18) = 3.47, *p* = .08, 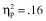; Group × Pressure, *F*(1,18) = 0.11, *p* = .75, 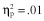. The grand mean percentage of putts holed was 66.92% (experts, *M* = 62.67%, *SD* = 14.51; novices, *M* = 71.16%, *SD* = 16.58). Thus, our manipulation of hole diameter (namely, 10.8 cm for novices and 5.4 cm for experts) was successful in ensuring that all participants holed a similar number of putts, with the overall success rate being less than 70%. This allowed us to consider putt outcome as a factor in our analyses (e.g., Babiloni et al., 2008).

#### Pressure

A 2 Group (expert, novice) × 2 Pressure (low, high) ANOVA for self-reported pressure confirmed main effects for both group, *F*(1,18) = 13.50, *p* < .01, 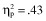, and pressure, *F*(1,18) = 11.10, *p* < .01, 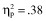, but no Group × Pressure interaction, *F*(1,18) = 0.39, *p* = .54, 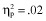. Pressure was greater in novices (*M* = 3.98, *SD* = 1.16) than in experts (*M* = 2.63, *SD* = 1.16), and increased from the low-pressure condition (*M* = 2.90, *SD* = 0.91) to the high-pressure condition (*M* = 3.70, *SD* = 1.04), thereby confirming that our pressure manipulation was successful.

### Cardiac and Muscle Activity

**Figure 1:**
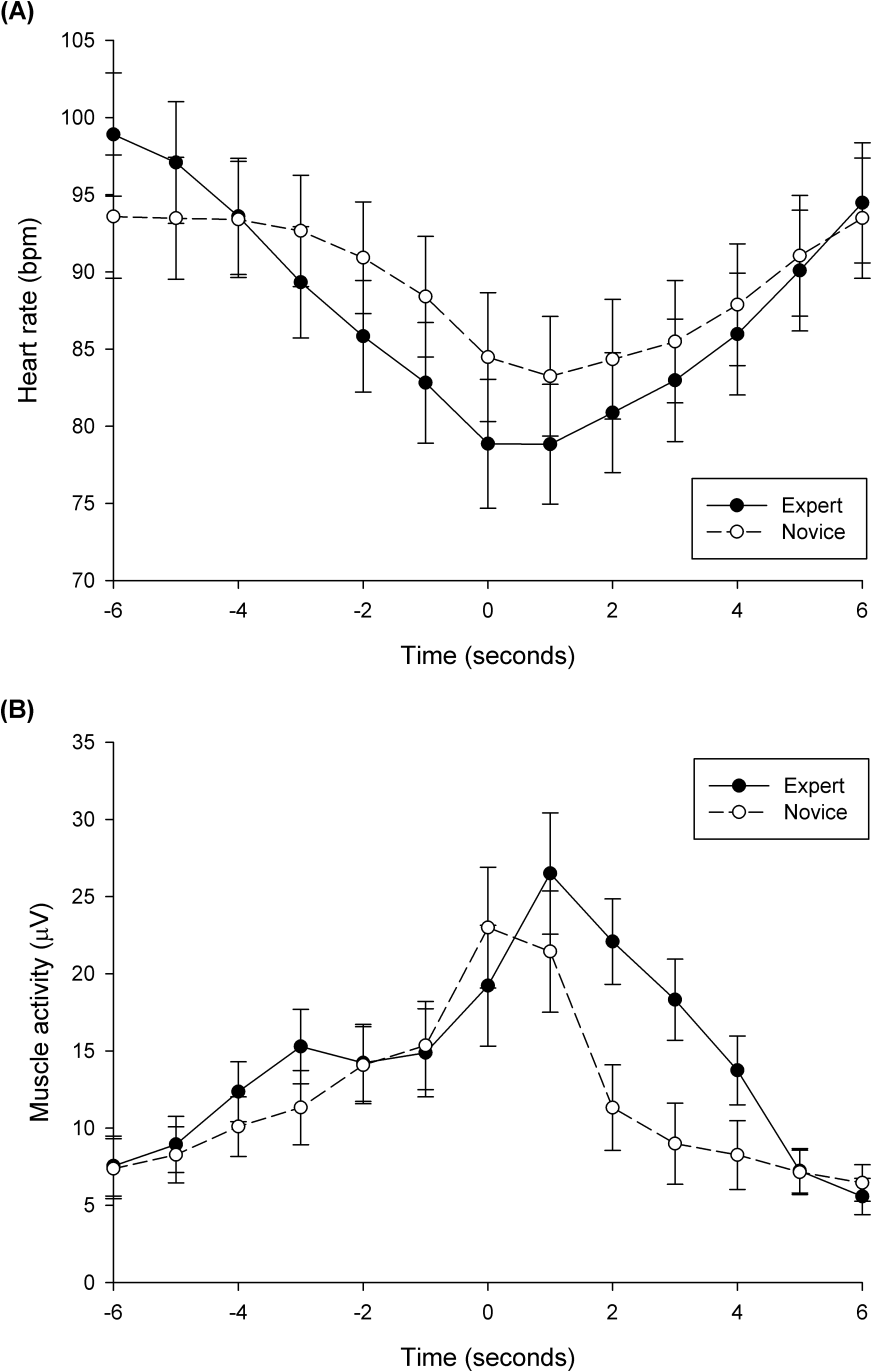
A: Heart rate Group × Epoch interaction. B: Muscle activity Group × Epoch interaction. Error bars indicate standard error of the means.

The results of 2 Group (expert, novice) × 2 Pressure (low, high) × 2 Outcome (hit, miss) × 13 Epoch (−6 s, −5 s, −4 s, −3 s, −2 s, −1 s, 0 s, +1 s, +2 s, +3 s, +4 s, +5 s, +6 s) ANOVAs performed on the measures of cardiac and muscle activity are illustrated in Figure 1. For heart rate, there were pressure, *F*(1,18) = 18.72, *p* < .001, 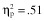, outcome, *F*(1,18) = 5.07, *p* < .05, 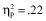, and epoch *F*(12,216) = 31.67, *p* < .001, 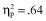, ε = .24, main effects as well as a Group × Epoch interaction effect, *F*(12,216) = 3.34, *p* < .05, 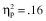, ε = .24. Heart rate was faster in the high-pressure condition (*M* = 90.48, *SD* = 12.20 bpm) than in the low-pressure condition (*M* = 86.60, *SD* = 11.33 bpm), and was somewhat higher for holed putts (*M* = 88.74, *SD* = 11.61 bpm) than missed putts (*M* = 88.34, *SD* = 11.61 bpm). The effect of epoch was best characterized by a quadratic polynomial trend (*p* < .001, 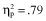). Finally, the Group × Epoch interaction reflected a group difference in the quadratic trend (*p* < .01, 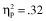), which was stronger in experts than in novices. In sum, the heart rate deceleration began earlier and was twice as pronounced in experts compared to novices (Figure 1A).

For muscle activity, there was a main effect of epoch, *F*(12,216) = 22.58, *p* < .001, 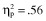, ε = .34 and a Group × Epoch interaction *F*(12,216) = 3.46, *p* < .05, 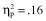, ε = .34. The effect of epoch was best characterized by a quadratic polynomial trend (*p* < .001, 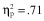). Finally, the Group × Epoch interaction reflected a group difference in the cubic trend (*p* < .05, 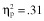), which was stronger in experts than in novices. This effect was further evidenced by *t* tests, which indicated that muscle activity was greater for the experts than novices at the +2 and +3 s epochs. In sum, experts maintained an increased level of muscle activity for a longer period after movement when compared to novices (Figure 1B).

### Cortical Activity

Separate 2 Group (expert, novice) × 2 Pressure (low, high) × 2 Outcome (hit, miss) × 5 Epoch (i.e., −4 to −3 s, −3 to −2 s, −2 to −1 s, −1 s to 0 s, 0 s to +1 s) ANOVAs were employed to examine the EEG measures. These analyses revealed no main effects of group or pressure. However, there were outcome and epoch main effects, as well as Group × Epoch and Outcome × Epoch interaction effects. The significant results are described below.

#### Epoch and group effects

Main effects of epoch were revealed at all sites and for all frequency bands (with the exception of low-alpha power at Cz), *F*s(4,72) = 2.48–17.42, *p*s < .05, 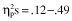, εs = .63–.91. These were best characterized by linear polynomial trends in the theta band and cubic polynomial trends in the other bands (*p*s < .05, 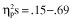).

**Figure 2:**
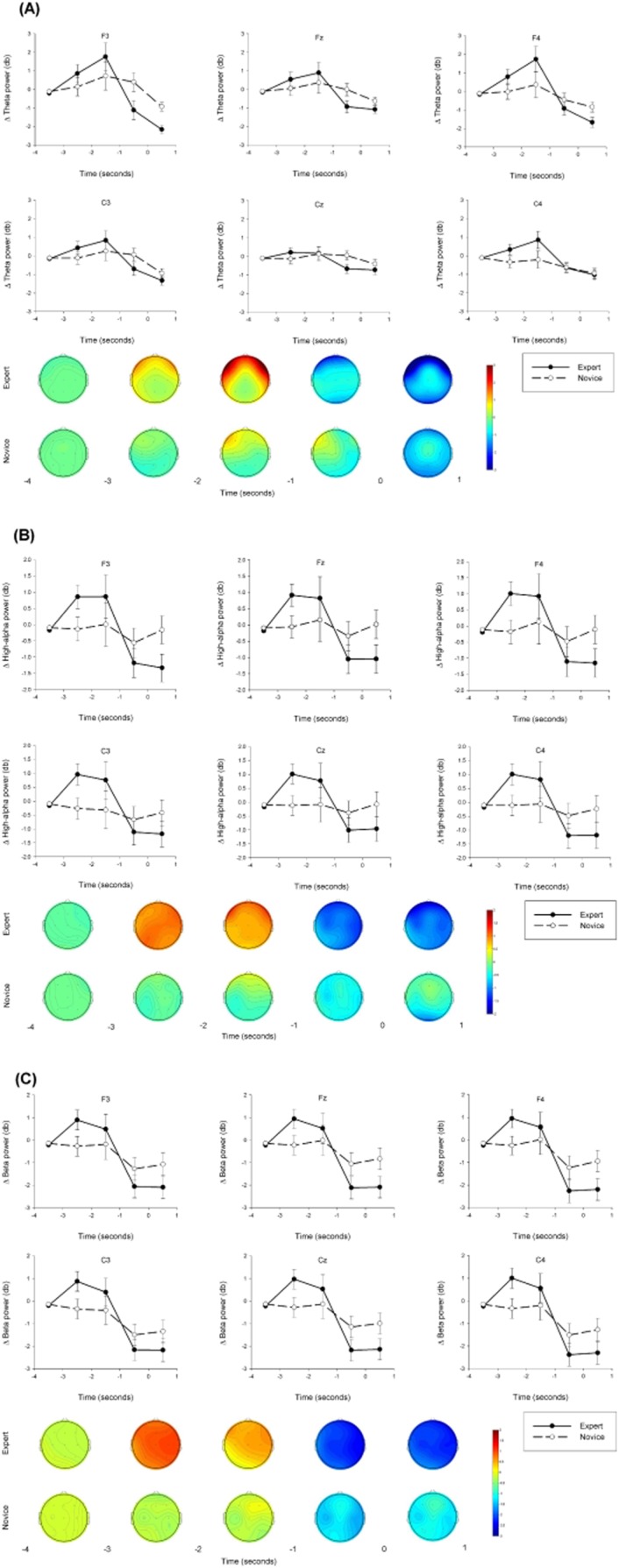
Line plots and topographical scalp maps to depict (A) theta power Group × Epoch interactions, (B) high-alpha power Group × Epoch interactions, and (C) beta power Group × Epoch interactions. Error bars indicate standard error of the means.

Group × Epoch interactions were revealed at the F3 and F4 sites in the theta power band, and at all sites in the high-alpha and beta power bands, *F*s(4,72) = 3.05–4.25, *p*s < .05, 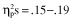, εs = .63–.91. These interactions reflected group differences in the linear trends (*p*s < .05, 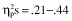), which were stronger in experts than in novices. These effects were further evidenced by *t* tests, which indicated that high-alpha and beta power were greater in experts than novices in the −3 s to −2 s epoch, while theta power was lower in experts than novices in the −1 s to 0 s and 0 s to 1 s epochs. The Group × Epoch interactions are illustrated in Figure 2.

#### Epoch and outcome effects

Outcome main effects were revealed at the Fz and F4 sites in the low-alpha power band, and at the Fz, F3, F4, and Cz sites in the high-alpha power band, *F*s(1,18) = 5.53–6.81, *p*s < .05, 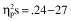. Holed putts were characterized by less low-alpha power (*M*s = −0.07–−0.09, *SD*s = 0.68–0.72 Δ power) and less high-alpha power (*M*s = −0.23–−0.32, *SD*s = 0.97–1.05 Δ power) than missed putts (low-alpha *M*s = 0.15–0.17, *SD*s = 0.69–0.72 Δ power; high-alpha *M*s = −0.06–0.07, *SD*s = 0.92–0.99 Δ power).

**Figure 3:**
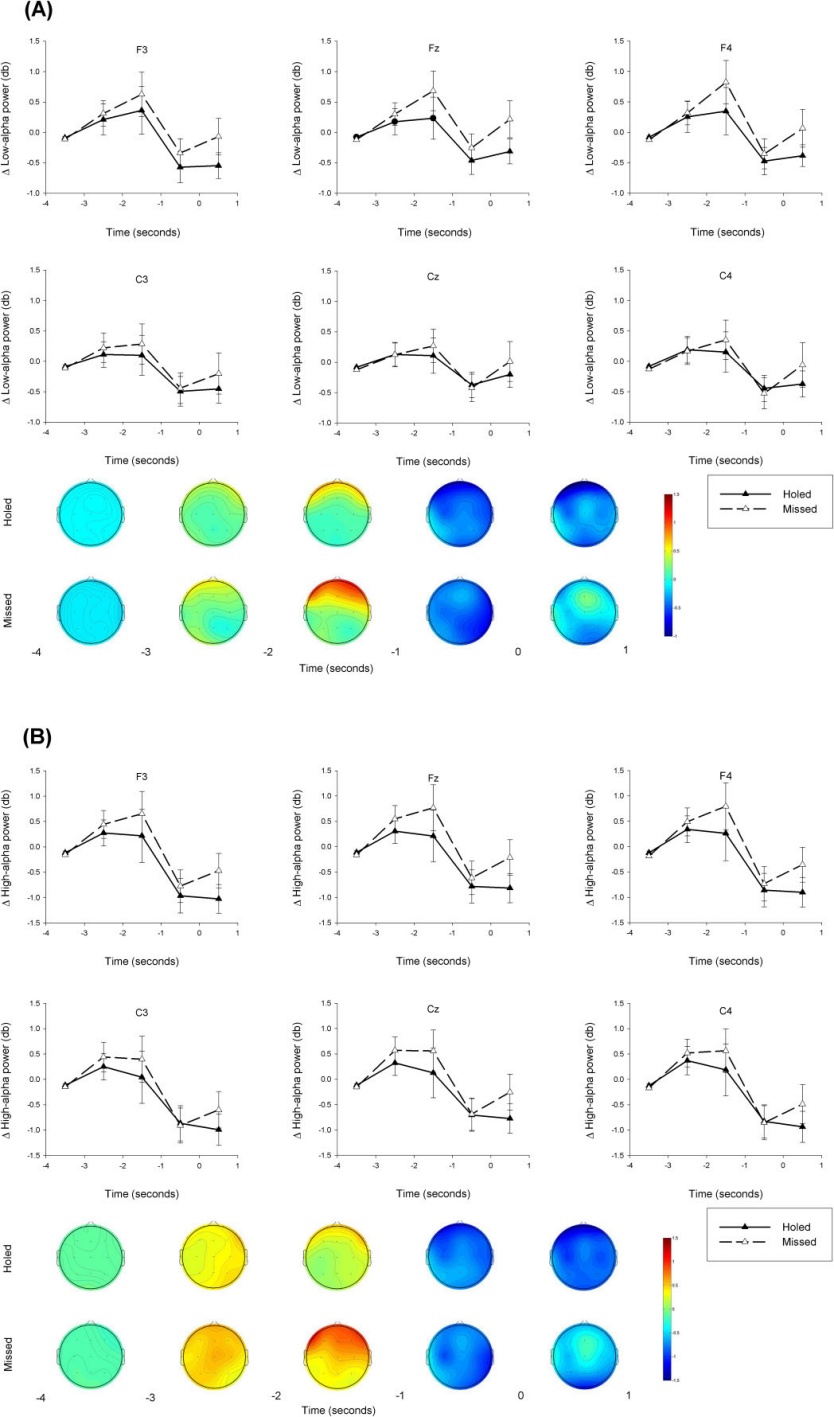
Line plots and topographical scalp maps to depict (A) low-alpha power Outcome × Epoch interactions, and (B) high-alpha power Outcome × Epoch interactions. Error bars indicate standard error of the means.

Outcome × Epoch interaction effects were revealed at the Fz and F4 sites in the low-alpha power band and at the Fz, F3, F4, and Cz sites in the high-alpha power band, *F*s(4,72) = 2.27–3.95, *p*s < .05, 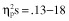. These interactions reflected outcome differences in the linear trends (*p*s < .05, 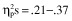), which were stronger for holed putts than missed putts. These effects were further evidenced by *t* tests, which indicated less low-alpha power and less high-alpha power for holed putts than missed putts in the −2 s to −1 s and 0 s to 1 s epochs. The Outcome × Epoch interactions are presented in Figure 3.

### Movement Kinematics

Two Group (expert, novice) × 2 Pressure (low, high) × 2 Outcome (hit, miss) ANOVAs employed to examine the kinematic measures revealed main effects of group for X-axis acceleration, *F*(1,18) = 30.83, *p* < .001, 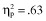 and impact velocity, *F*(1,18) = 10.44, *p* < .01, 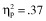. *T* tests indicated that X-axis (i.e., lateral) clubhead acceleration and impact velocity were greater in novices (X-axis acceleration *M* = 0.44, *SD* = 0.10 m.s^−2^; impact velocity *M* = 0.57, *SD* = 0.24 m.s^−1^) than in experts (X-axis acceleration *M* = 0.26, *SD* = 0.10 m.s^−2^; impact velocity *M* = 0.33, *SD* = 0.24 m.s^−1^). In sum, experts swung more slowly and maintained the club in a more linear back-and-forth plane (i.e., were less likely to swing out of line) when compared to novices.

## Discussion

There are several enticing incentives, such as the possibility of expedited motor skill acquisition, which could be harnessed through research into the movement-related psychophysiological response patterns that characterize successful motor performance (Thompson, Steffert, Ros, Leach, & Gruzelier, 2008). This experiment was designed to extend previous studies by providing the first multimeasure assessment of the psychophysiology of optimal performance under both low- and high-pressure conditions. Our results are discussed in the sections that follow.

### Movement-Related Psychophysiological Activity as a Function of Expertise

In line with our hypotheses, we found that golf putts were preceded by a deceleration in heart rate, which was greater in experts than novices. Specifically, experts slowed their heart rates by 20 bpm, whereas novices slowed theirs by 9 bpm in the 6 s preceding movement onset. We also found a widespread reduction in EEG high-alpha power that tended to be greater in experts than novices. Specifically, experts reduced EEG high-alpha power by 1.0 decibel compared to a reduction of just 0.4 decibels for novices in the 4 s preceding movement. However, an interesting caveat to the latter finding was that the Group × Epoch interaction for high-alpha power was mainly driven by experts displaying more high-alpha power than novices in the early phases of movement preparation (i.e., 2 s–3 s before movement, see Figure 2B). This could indicate that experts are more relaxed and expend fewer cortical resources than novices upon addressing the ball (e.g., Pfurtscheller, 1992). The clear reduction in high-alpha power that subsequently occurs could then reflect experts focusing their attention and mobilizing resources to program the force and direction of putts during the final 2 s preceding movement (e.g., Pfurtscheller & Aranibar, 1979).

Accordingly, when taken together, these findings can be interpreted to indicate that expert golfers engage in more external information processing (e.g., Lacey & Lacey, 1974), and devote more neural resources to the response programming of golf putts (e.g., Pfurtscheller, 1992) during the final seconds of preparation for action. For instance, it is possible that experts focus more attention on the ball (e.g., Moore et al., 2012), and utilize their greater bank of previous experiences stored in working memory to actively inform the programming of movement direction and force (cf. Neubauer & Fink, 2009).

In addition to these hypothesized findings, we uncovered further effects of expertise on cortical activity outside of the high-alpha power band. For instance, putts were preceded by a reduction in theta power that was greater in experts than novices. Reduced theta power has been associated with an increase in focused attention (e.g., Bakhshayesh, Hansch, Wyschkon, Rezai, & Esser, 2011), and thus could further reflect experts allocating more attention and expending more neural resources than novices during the final moments of preparation for putts (e.g., Milton, Solodkin, Hlustík, & Small., 2007).

Similarly, we found a widespread reduction in beta power, which also tended to be greater in experts than novices. However, as was the case in the high-alpha power band, this Group × Epoch interaction was mainly driven by experts displaying more beta power than novices in the early phases of movement preparation (i.e., 2 s–3 s before movement, see Figure 2C). Such synchronization of beta power occurring around voluntary self-paced movements has been associated with reduced excitability of motor cortex neurons (e.g., Chen, Yassen, Cohen, & Hallett, 1998). This further implies that experts are more relaxed and expend fewer cortical resources than novices during the early phases of motor preparation. However, this trend soon reverses as experts are able to more accurately program parameters such as force and direction during the final 2 s preceding movement.

Aside from these effects on cardiac and cortical variables, experts and novices also produced different patterns of muscle activity. Specifically, experts displayed greater muscle activity than novices in the seconds immediately following putts. Because increased muscle activity in experts occurred after putts had been struck, this effect is unlikely to reflect differences in preparatory information processing and response programming. Instead, it could reflect a difference in technique between experts and novices, such as experts maintaining their form by holding the putter in its end position for a few seconds at the conclusion of each putting stroke (cf. Delay, Nougier, Orliaguet, & Coello, 1997).

Finally, analyses of our kinematic variables revealed that the movement-related psychophysiological responses of experts were accompanied by more favorable movement patterns, as reflected by their striking the ball more slowly and being less likely to swing out of line, when compared to novices (e.g., Delay et al., 1997; Sim & Kim, 2010). This means that experts were less likely than novices to significantly overshoot the hole or push and pull putts wide of the hole.

In sum, an integrated view of our findings could point to the following chain of events. First, based on their greater heart rate deceleration, experts process pertinent environmental stimuli to a greater extent than novices. Second, an initial consequence of this external focus is greater mental relaxation, which is reflected by increased theta, high-alpha, and beta power up until 2 s before movement initiation. Third, as external information processing increases (e.g., around the time of maximal bradycardia), we can speculate that experts allocate more cortical resources to response programming as reflected by the dynamic reduction in theta, high-alpha, and beta power during the final 2 s of motor preparation. Finally, it appears that this pattern of preparatory activity collectively helps to produce a more favorable putting technique, as manifested by a more linear swing.

### Movement-Related Psychophysiological Activity as a Function of Performance Outcome

Our hypothesis that holed putts would be preceded by a greater heart rate deceleration than missed putts was not supported (i.e., there was no Outcome × Epoch interaction effect). This finding is in line with the notable absence of accuracy-related differences in heart rate deceleration in the preparation for action literature (e.g., Konttinen et al., 1998). It is important to recognize that autonomic variables can only provide an indirect window into cognitive/attentional processes (e.g., Hugdahl, 1996). Thus, preparatory heart rate deceleration may not be a good enough measure of attentional focus to reliably predict performance outcomes. In spite of the null Outcome × Epoch interaction for heart rate, it should be noted that there was an outcome main effect whereby heart rate was marginally faster during the seconds surrounding putts that were holed compared to those that were missed. This difference of less than 1 bpm is likely to have been driven by an excitatory response associated with processing the successful outcome of holed putts.

Finally, our results did provide support for the hypothesis that holed putts would be preceded by less EEG alpha power than missed putts. This finding, which is in line with the results of Babiloni et al. (2008), was especially evident during the window around the initiation of movement (2 s preceding until 1 s after), and at frontal and central sites (i.e., Fz, F3, F4, Cz) that roughly overlie motor areas of the cerebral cortex (Ashe et al., 2006). Accordingly, these results could further reflect more neural resources being devoted to the accurate programming of movement force and direction ahead of putts that were holed (e.g., Pfurtscheller & Aranibar, 1979).

### Movement-Related Psychophysiological Activity as a Function of Pressure

We supported our hypothesis that increased pressure would elicit an increase in heart rate. Importantly, however, these results were not associated with pressure-induced impairments in putting performance, as percentage of putts holed did not differ between the low- and high-pressure conditions. Similarly, pressure elicited no effects on any of the other psychophysiological or kinematic variables. This result is not surprising when interpreted alongside the null effect of pressure on performance.

The lack of pressure effects could be attributed to the high number of trials that were required to generate meaningful EEG data (e.g., Luck, 2005). Specifically, it is known that multiple trials dilute the strength of pressure manipulations, in this case providing participants with several chances to redeem bad putts (cf. Cooke et al., 2010; Woodman & Davis, 2008). It is recommended that future studies afford special consideration to methods of maximizing the potency of their pressure manipulations, especially where large numbers of trials are planned.

### Limitations and Directions for Future Research

Our results should be interpreted in light of some methodological limitations. First, we did not measure ventilation. We have no reason to suspect that ventilation patterns would have differed between the various factors included in our experiment (e.g., Boutcher & Zinsser, 1990). Nonetheless, such measures could offer an even more detailed understanding of patterns of cardiac deceleration preceding movement (e.g., Mulder, 1992).

Second, our sample size was relatively small. This was due to few golfers meeting our stringent inclusion criterion of having a golf handicap < 5. However, our sample size is similar to or larger than the sample sizes adopted by relevant previous studies (e.g., Baumeister et al., 2008, *N* = 18; Babiloni et al., 2008, *N* = 12; Sim & Kim, 2010, *N* = 10). Moreover, our study was sufficiently powered to detect a number of main and interaction effects as detailed above. Notwithstanding, it may be beneficial for future studies to replicate and extend our experiment with a larger cohort.

Third, it should be conceded that the sensitivity of our performance measure may not have matched the sensitivity of our psychophysiological measures. For instance, overstruck putts may have fortuitously hit the lip of the hole and dropped in, particularly among the novices who putted to a larger hole than the experts. To gain a cleaner picture of the differences in response programming between successful and unsuccessful trials, future studies could ask participants to putt to a smooth target and obtain more precise measures of error in both length and direction (e.g., Hancock, Butler, & Fischman, 1995).

Relatedly, future research aiming to provide a richer insight into the psychophysiological predictors of successful performance outcomes could consider the effects of trial history. Indeed, if the amount of resources devoted to response programming relates positively to putting accuracy, it is tempting to speculate that the best performers will be quicker at detecting errors after bad putts, and mobilize more resources in an attempt to eradicate the error during preparation for subsequent putts (e.g., Manoach et al., 2007; Sherwin & Sajda, 2013). These hypotheses could be tested by examining after putting error-related negativity and preparatory high-alpha power as a function of whether the previous trial was holed or missed.

Finally, it would be interesting for future research to use the results yielded in the present study to inform biofeedback interventions. In brief, biofeedback training provides individuals with real-time information about their levels of physiological activity via sounds or visual displays (Hammond, 2007). Based on principles derived from operant learning theory (e.g., Skinner, 1963), rewarding positive reinforcement, such as a change in the pitch of a tone, is provided when a desired level of physiological activity is achieved. The results of the present experiment suggest that a biofeedback intervention focused on teaching golfers to produce a dynamic reduction in frontal and central high-alpha power before initiating putts could expedite the evolution from novice to expert and increase the likelihood of putting success.

### Conclusion

In conclusion, this experiment provides the most compelling evidence to date to indicate that a greater heart rate deceleration and a greater reduction in EEG theta, high-alpha, and beta power in the final seconds preceding golf putts are associated with greater expertise. Moreover, premovement EEG high-alpha power emerged as a key variable that was predictive of both expertise and successful performance outcomes, with participants producing less high-alpha power in the seconds around putts that were holed when compared to those that were missed. These effects could pave the way for biofeedback studies that train participants to reduce high-alpha power in the final moments preceding golf putts or similar self-paced accuracy tasks. Such studies promise to yield highly exciting developments for the motor learning protocols of the future.
